# Apelin-17 to diagnose idiopathic pulmonary arterial hypertension: A biomarker study

**DOI:** 10.3389/fphys.2022.986295

**Published:** 2023-01-04

**Authors:** Vasile Foris, Gabor Kovacs, Alexander Avian, Zoltán Bálint, Philipp Douschan, Bahil Ghanim, Walter Klepetko, Andrea Olschewski, Horst Olschewski

**Affiliations:** ^1^ Division of Pulmonology, Department of Internal Medicine, Medical University of Graz, Graz, Austria; ^2^ Ludwig Boltzmann Institute for Lung Vascular Research, Graz, Austria; ^3^ Institute for Medical Informatics, Statistics and Documentation, Medical University of Graz, Graz, Austria; ^4^ Faculty of Physics, Babes-Bolyai University Cluj-Napoca, Cluj-Napoca, Romania; ^5^ Division of General and Thoracic Surgery, University Hospital Krems, Karl Landsteiner University of Health Sciences, Krems an der Donau, Austria; ^6^ Division of Thoracic Surgery, Department of Surgery, Medical University of Vienna, Vienna, Austria; ^7^ Experimental Anesthesiology, Department of Anesthesiology and Intensive Care Medicine, Medical University of Graz, Graz, Austria

**Keywords:** apelin-17, NT-proBNP, GDF-15, blood derived biomarker, diagnosis, idiopathic pulmonary arterial hypertension

## Abstract

**Background:** NT-proBNP and GDF-15 are established blood-derived biomarkers for risk assessment in pulmonary hypertension (PH), despite limited sensitivity and specificity. Apelin has a crucial function in endothelial homeostasis, thus it might represent a new biomarker for PH. However, there are numerous circulating apelin isoforms, and their potential role in this setting is unknown. This study evaluated different apelin isoforms in PH patients and prospectively evaluated the role of apelin-17 in comparison with NT-proBNP and GDF-15 as diagnostic marker in idiopathic pulmonary arterial hypertension (IPAH).

**Methods:** Based on our pilot study, we performed a power calculation for apelin-13, apelin-17, apelin-36, as predictor of IPAH vs healthy controls. Apelin-17 provided the best discriminatory power, and accordingly, we enrolled n = 31 patients with IPAH and n = 31 matched healthy controls in a prospective study. NT-proBNP and GDF-15 was determined in all patients. ROC curve analysis was performed to assess the diagnostic value of the markers and their combinations.

**Results:** Apelin-17, NT-proBNP, and GDF-15 were significantly elevated in IPAH patients as compared to controls (*p* < .001). Apelin-17 detected IPAH with a sensitivity of 68% and a specificity of 93% at a cut-off value of >1,480 pg/ml (AUC 0.86, 95%CI:0.76–0.95) as compared to GDF-15 (sensitivity 86%; specificity 72%, AUC 0.81 (95%CI:0.7–0.92)) and NT-proBNP (sensitivity 86%; specificity 72% (AUC 0.85, 95%CI:0.75–0.95)). Combinations of these markers could be used to increase either specificity or sensitivity.

**Conclusion:** Apelin-17 appears to be suitable blood derived diagnostic marker for idiopathic pulmonary arterial hypertension.

## 1 Introduction

A biomarker aims to improve prognosis prediction or risk assessment or to be of diagnostic value. In pulmonary hypertension (PH) patients several blood-derived biomarkers have been predictive for prognosis e.g. creatinine, troponins, red cell distribution width, GDF-15, endostatin, free fatty acids, YKL-40, and uric acid to natriuretic peptides (Torbicki et al., 2003; Leuchte et al., 2007; Njaman et al., 2007; Nickel et al., 2008; Shah et al., 2008; Rhodes et al., 2011; Heresi et al., 2012; Chen et al., 2014; Damico et al., 2015; Simpson et al., 2019; Geenen et al., 2020; Huai et al., 2021; Hendriks et al., 2022), howeverin inthe international guidelines only NT-proBNP and BNP are recommended. (Humbert et al., 2022). Biomarkers that have a unique diagnostic value are missing. However, there are reasons to beleieve that apelin is a promising candidate.

There is growing evidence that the APJ/apelin axis, closely related to the ACE2 system, plays an essential role in maintaining pulmonary vascular endothelial cell homeostasis by positively influencing survival, proliferation, and migration (Alastalo et al., 2011). APJ belongs to the G-protein coupled receptors, and its role in the pulmonary circulation has been extensively reviewed (Andersen et al., 2011; Yang et al., 2015; Chatterjee et al., 2020; Liu et al., 2020). The APJ receptor is abundant in the pulmonary vasculature on endothelial and smooth muscle cells. Its endogenous ligand, apelin, is a promising candidate biomarker for PH. However, it has at least 46 isoforms with different biological functions (Mesmin et al., 2011).

The apelin gene encodes the 77-amino acid preproapelin, cleaved at its C-terminus to produce the 55-amino acid prohormone apelin-55. The intracellular processing of apelin-55 results in many bioactive residues circulating in the body. Although they could all be potential biomarkers for PH, there is limited data available. In most of these studies, only one isoform was measured, either apelin-13 or apelin-12, and the cohorts were small and quite heterogeneous. Chandra et al. found that apelin-12 levels were lower in n = 23 PH patients than in controls (Chandra et al., 2011). These data demonstrate that disruption of apelin signaling can exacerbate PH mediated by decreased activation of AMP-activated kinase and eNOS, and they identify this pathway as a potentially important therapeutic target for treatment of this refractory human disease. Others found reduced apelin-36 plasma levels in patients with parenchymal lung diseases and PH patients (Goetze et al., 2006). However, there is no data on the diagnostic value of other circulating apelin isoforms and the studies were not designed to evaluate apelin as a diagnostic biomarker for PH. Particularly apelin-17 appears to be of interest, as this isoform has the highest binding potency to the APJ receptor (Yang et al., 2017a; Read et al., 2020).

We prospectively investigated apelin-17 as a diagnostic biomarker in IPAH patients compared to matched controls and found that the accuracy in PH detection was comparable to NT-proBNP and GDF-15.

## 2 Methods

### 2.1 Prospective study

In our prospective study, we enrolled a total of n = 31 IPAH patients from our pulmonary hypertension clinic and n = 31 age- and sex-matched healthy controls between January 2013 and January 2014. IPAH was diagnosed if the patient met the hemodynamic criteria: mean pulmonary arterial pressure (mPAP) ≥ 25 mmHg, pulmonary vascular resistance (PVR)> 3WU, pulmonary arterial wedge pressure (PAWP) ≤ 15 mmHg and relevant diseases of the left heart, lung, liver, kidney, and blood as well as perfusion defects on the lung perfusion scan were excluded. The other inclusion criteria were a written informed consent for the study and for the storage of blood samples in the biobank, age >18 years and no clinical signs of acute cardiac decompensation.

Exclusion criteria were withdrawal of consent or evidence of significant left heart, lung, liver, kidney or blood disease. Peripheral blood was collected from an antecubital vein into 9 ml Vacutainer^®^ tubes with separating gel. After the initial 21 IPAH patients and their age- and sex-matched controls (Supplementary Figure S2 in the Supporting Information), due to slow recruitment, we enrolled the same 10 IPAH patients and 10 controls who had already participated in the pilot study, on a *de novo* basis. This adaptive design allowed us to include a sufficient number of patients in a reasonable period of time (Leon et al., 2011; Whitehead et al., 2016). All samples were reversibly anonymized, and investigators were blinded to clinical data. The primary endpoint was the accuracy of apelin-17 to discriminate IPAH patients from controls. In addition, we exploratively included all eligible CTEPH patients during the enrollment period and tested the same potential biomarkers. Subjects gave written informed consent, and the study was approved by the local ethics committee (23–408 ex 10/11).

Diagnostic workup of IPAH and CTEPH patients was performed according to the ERS/ESC guidelines and the protocol of the sixth World Symposium for Pulmonary Hypertension proceedings (Simonneau et al., 2019; Humbert et al., 2022). Right heart catheterization was performed on a regular basis when pulmonary hypertension was suspected based on unexplained dyspnoea or the results of transthoracic Doppler echocardiography, ECG, or thoracic imaging. Throughout the acquisition period, the same experienced team performed diagnostic procedures in a standardized manner. Patients were usually admitted 1 day prior to the RHC, where the actual blood tests, ECG, and chest X-ray were performed. Diagnostic workup included high-resolution computed tomography of the chest, lung perfusion scintigraphy, pulmonary function tests and exercise testing. All subjects gave written informed consent for the clinical procedures. For the right heart catheter investigation after local anaesthesia, the jugular vein was approached using the Seldinger technique. A balloon-tipped Swan-Ganz catheter was inserted until the wedge position was achieved. The zero reference line was placed at the midthoracic level (Kovacs et al., 2014).

### 2.2 Biomarker assessment

Serum samples were stored at room temperature for 30 min, then centrifuged at 1500 G for 10 min and aliquoted to 250 µl. All samples were stored at -80°C in the Biobank of the Medical University of Graz until analysis. For the determination of apelins and GDF-15, commercially available enzyme-linked immunosorbent assays (ELISA) were used (apelin-13: Cusabio Biotech Co., LTD., Cat. No. CSB-E13072h), (apelin-17: Antibodies online Cat. No. ABIN6953820 for the pilot study and USCN Life Science Inc., Cat. No. CED065Hu for the prospective study), (apelin-36: Cusabio Biotech Co., LTD., Cat. No. CSB-E13566h), (GDF-15: R&D, Cat. No. DGD150). According to the manufacturer, no significant cross-reactivity or interference was observed between apelin-17 and analogues. NT-proBNP was determined during routine testing at the hospital’s central laboratory (Roche, Cobas).

### 2.3 Pilot study

To test the utility of apelins as possible biomarkers for PH, we performed a pilot study with 10 IPAH patients and 10 matched controls, in which we examined serum levels of apelin-12, apelin-13, apelin-17, apelin-36, NT-proBNP, and GDF-15 (Foris et al., 2013). We found that several apelin isoforms were elevated in IPAH, with apelin-17 having the best discriminatory power (Supplementary Figure S1 in the Supporting Information). Apelin-12 was below the limit of detection in most patients and was therefore not further investigated.

### 2.4 Statistical analysis

The power analysis for apelin-17, providing 80% discriminatory power for detecting differences at an alpha level of 0.05, yielded a sample size of n = 31 IPAH patients and n = 31 healthy controls. Data are presented as means ± standard deviation or median and interquartile range (IQR). Statistical analysis was performed using GraphPad Prism software (Version 5.04, GraphPad Software Inc., La Jolla, California) and SPSS (Version 23, SPSS Inc., Chicago, IL, USA). For the analysis of differences between groups, Kruskal–Wallis test was used followed by Dunn´s multiple comparison test. To analyse the association between parameters, Pearson correlation analysis was used for normally distributed values and Spearman correlation for non-normally distributed values. ROC analysis was performed to determine the diagnostic value of apelin-13, apelin-17, apelin-36, GDF-15 and NT-proBNP. The Area Under the Curve (AUC) and corresponding 95% confidence intervals were calculated. The best cut-off score was calculated according to the Youden index. *p* < 0.05 was considered statistically significant.

## 3 Results

Patient characteristics of the N = 31 enrolled IPAH patients, with their age- and sex-matched healthy controls and the 24 CTEPH patients are shown in Table 1.

**TABLE 1:** Patients’ characteristics. Values are expressed as mean ± standard deviation (mean ± SD) or median and interquartile range (median (IQR)).

	Control (n = 31)	IPAH (n = 31)	CTEPH (n = 24)
Age (years) (mean ± SD)	55.9 ± 17.1	56.8 ± 17	65 ± 16.7
Sex (F/M)	23/8	23/8	15/9
mPAP (mmHg) (mean ± SD)	NA	49.2 ± 18.7	40.7 ± 13.7
RAP (mmHg) (mean ± SD)	NA	7 ± 4	8 ± 4
PAWP (mmHg) (mean ± SD)	NA	9 ± 4	9 ± 3
PVR (WU) (median (IQR))	NA	7.9 (4.6–14)	8.5 (5.1–11.7)
CI (L/min/m^2^) (mean ± SD)	NA	2.5 ± 0.8	2.3 ± 0.5
6MWD (m) (median (IQR))	NA	400 (322–455)	405 (273–448)
WHO class (I/II/III/IV)	NA	0/13/15/3	1/11/12/0
NT-proBNP (pg/ml) (median (IQR))	84 (42–227)	997.5 (186–2092)	1,120 (320–2,333)
GDF-15 (pg/ml) (median (IQR))	497.1 (384–882.9)	1798 (857.1–3,430)	1,355 (600.1–2,474)
Apelin-17 (pg/ml) (median (IQR))	921 (746.8–1,182)	1,591 (1,247–2,245)	1,561 (909.2–1862)
Apelin-13 (pg/ml) (median (IQR))	245.6 (172.2–334.8)	212.3 (104.6–350.6)	326.4 (135.1–455.7)
Apelin-36 (pg/ml) (median (IQR))	252.8 (123.6–351)	179.3 (89.59–272.8)	523 (335–541.8)

F = female, M = male, mPAP = mean pulmonary arterial pressure, RAP = right atrial pressure, PAWP = pulmonary arterial wedge pressure, PVR = pulmonary vascular resistance, WU = Wood units, 6MWD = 6-min walk distance, WHO FC = World Health Organization functional class, NT-proBNP = N-terminal pro-brain natriuretic peptide, CTEPH = chronic thromboembolic pulmonary hypertension, IPAH = idiopathic pulmonary arterial hypertension, NA = not available.

### 3.1 Serum levels of biomarkers

**FIGURE 1:**
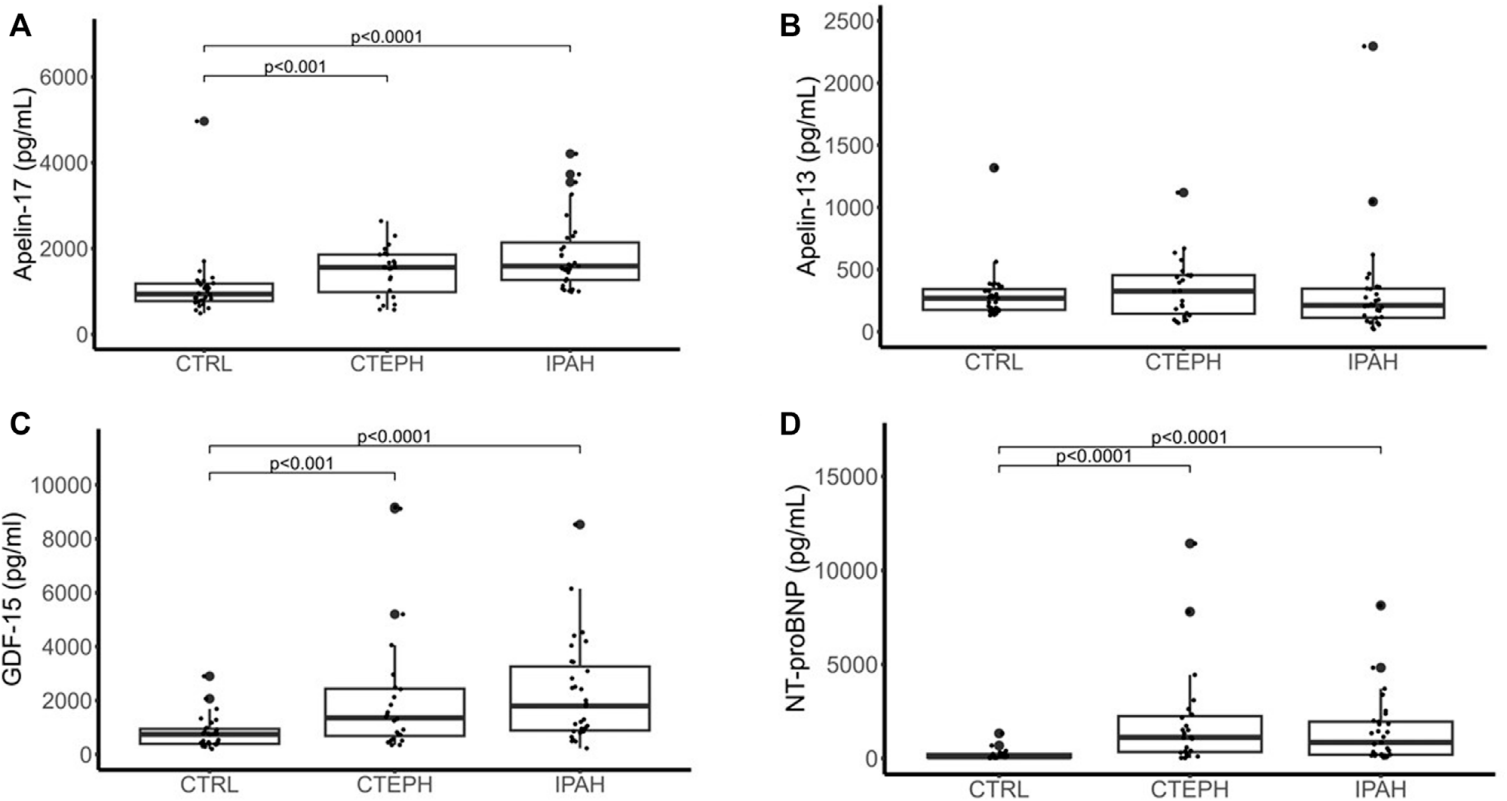
Serum levels of apelin-17 **(A)**, apelin-13 **(B)**, GDF-15 **(C)** and NT-proBNP **(D)** in n = 31 IPAH patients, n = 31 controls as well as n = 24 CTEPH patients represented as Whisker-Blot plots. Points above or below the plots represent outliers. *p* < 0.05 was considered statistically significant.

Apelin-17 (minimum detectable dose: 48.2 pg/ml) was detectable in all samples, except for one healthy control, and levels were elevated in both IPAH and CTEPH, compared to controls (IPAH vs CTRL vs CTEPH: median = 1591 pg/ml (IQR: 1,247–2,245) vs 921 pg/ml (IQR: 746.8–1,182) vs 1561 pg/ml (IQR: 909.2–1862), *p* < 0.0001 for IPAH vs control and *p* < 0.001 for CTEPH vs control, Figure 1A) with no significant differences between IPAH and CTEPH. Apelin-13 was detectable in all but one IPAH patient and showed no differences between the groups (Figure 1B).

GDF-15 (mean minimum detectable dose: 2 pg/ml) was detectable in all patients and turned out to be increased in both IPAH and CTEPH as compared to control (IPAH vs CTRL vs CTEPH: median = 1798 pg/ml (IQR: 857–3,430) vs 497 pg/ml (IQR: 384–883) vs 1,355 pg/ml (IQR: 600–2,474); IPAH vs CTRL *p* < 0.0001, CTEPH vs CTRL *p* < 0.001, Figure 1C), with no significant differences between CTEPH and IPAH.

NT-proBNP (normal range: less than 150 pg/ml) was not available in two IPAH patients, one CTEPH patient and six healthy controls and turned out to be increased in both IPAH and CTEPH as compared to control (IPAH vs CTRL vs CTEPH: median = 997.5 pg/ml (IQR: 186–2092) vs 84 pg/ml (IQR: 42–227) vs 1,120 pg/ml (IQR: 320–2,333); IPAH vs CTRL *p* < 0.0001, CTEPH vs CTRL *p* < 0.0001, Figure 1D), with no significant differences between CTEPH and IPAH.

Apelin-36 (minimum detectable dose: 14.2 pg/ml) turned out to be undetectable in a relatively large number of subjects (n = 7 controls, n = 2 IPAH and n = 16 CTEPH). If undetectable values are neglected, there was a significant difference between CTEPH and IPAH (CTEPH vs IPAH: 523 pg/ml (IQR: 335–542) vs 179 pg/ml (IQR: 90–273) *p* < 0.05), but this result does not allow to draw any conclusions.

### 3.2 Diagnostic value of biomarkers

**FIGURE 2:**
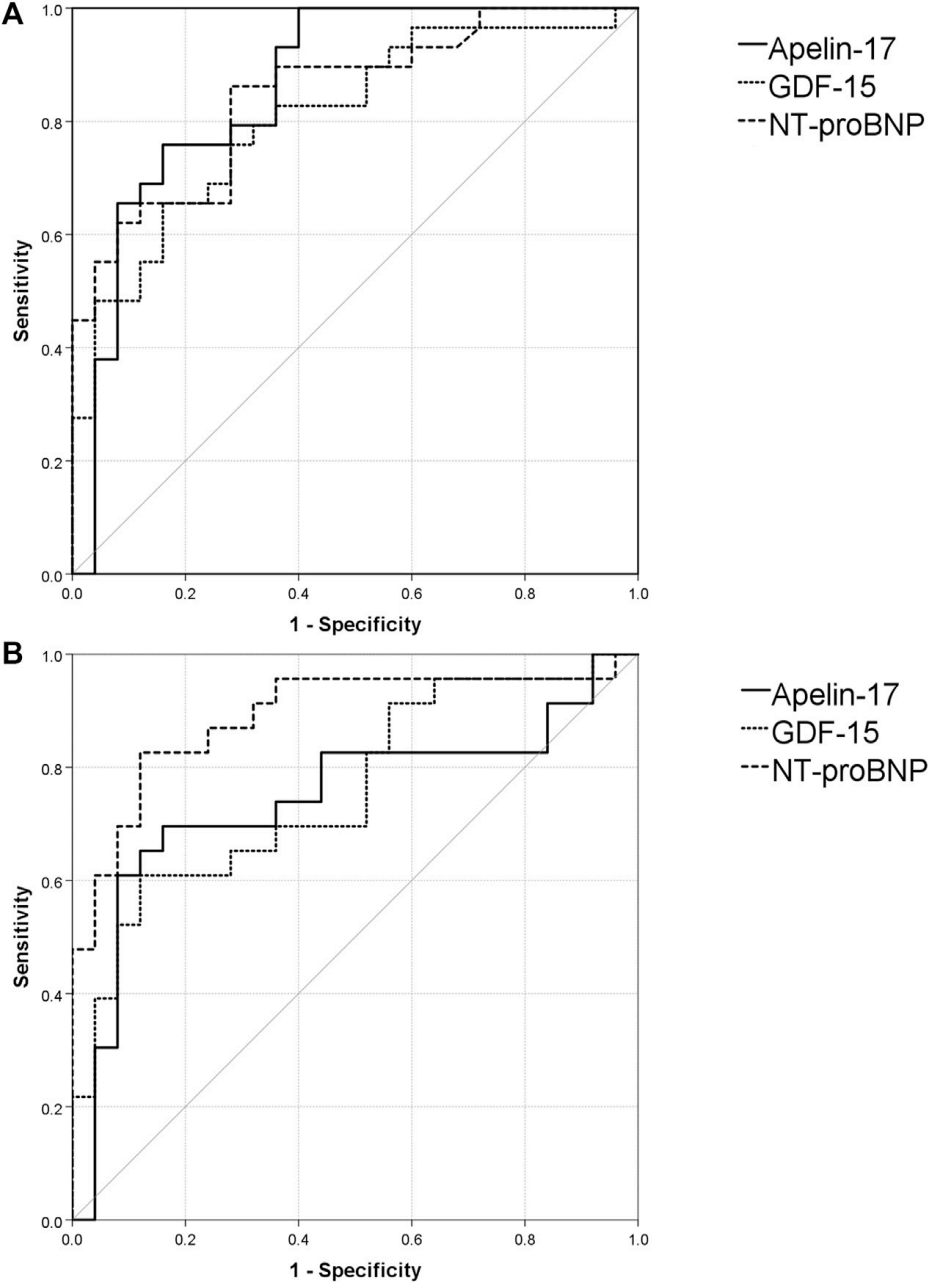
Receiver operating curves and area under the curve analysis of the investigated markers. Values of sensitivity (%) are represented on the *Y* axis. *X* axis represents 100% - specificity (%). **(A)**. ROC curves for apelin-17, GDF-15 and NT-proBNP for IPAH **(B)**. ROC curves for apelin-17, GDF-15 and NT-proBNP for CTEPH.

To assess the diagnostic accuracy of our markers in predicting IPAH compared with controls, we calculated the area under the curve (AUC) for the receiver operating curve (ROC). Apelin-17 distinguished IPAH from controls with an AUC of 0.86 (95%CI: 0.76–0.95)). At a cut-off value of 1,480 pg/ml, IPAH was detected with a sensitivity and specificity of 68% and 93%, respectively. Apelin-13 and apelin-36 did not predict IPAH. GDF-15 detected IPAH with an AUC of 0.81 (95%CI: 0.7–0.92). At a cut-off value of 855 pg/ml, sensitivity and specificity were 86% and 72%, respectively (Figure 2 A). NT-proBNP detected IPAH with an AUC of 0.84 (95%CI: 0.75–0.95) and at a cut-off value of 140 pg/ml, the sensitivity and specificity were 86% and 72%, respectively (Table 2).

**TABLE 2:** Apelin-17, GDF-15 and NT-proBNP for diagnosing IPAH and CTEPH.

	IPAH					CTEPH				
	AUC	*p*-value	Best cut-off	Sensitivity	Specificity	AUC	*p*-value	Best cut-off	Sensitivity	Specificity
Apelin-17	0.86 (0.76–0.95)	<0.001	>1,480	68%	93%	0.75 (0.56–0.89)	0.002	>1,270	71%	87%
GDF-15	0.81 (0.7–0.92)	<0.001	>855	77%	71%	0.73 (0.59–0.87)	0.004	>1,220	58%	84%
NT-proBNP	0.85 (0.75–0.95)	<0.001	>140	86%	72%	0.89 (0.79–0.99)	<0.001	>305	83%	88%

In CTEPH, apelin-17 (AUC 0.75, 95%CI: 0.60–0.89; best cut-off: >1,270 pg/ml; sensitivity 71%; specificity 87%) and apelin-36 (AUC: 0.84, 95%CI: 0.88–1.00; best cut-off: >320 pg/ml; sensitivity 86%; specificity: 77%) distinguished CTEPH from healthy controls. GDF-15 distinguished CTEPH from controls with an AUC of 0.73 (95%CI: 0.59–0.87) and at a cut-off of 1,220 pg/ml, sensitivity and specificity were 58% and 84%, respectively (Figure 2B). NT-proBNP distinguished CTEPH from controls with an AUC of 0.89 (95%CI: 0.79–0.99) and at a cut-off of 305 pg/ml, the sensitivity and specificity were 83% and 88%, respectively.

A model was used to combine markers to increase sensitivity or specificity. Three positive criteria, apelin-17 > 1,480 pg/ml, NT-proBNP >140 pg/ml, and GDF-15 > 855 pg/ml, in the same patient, were associated with a specificity of 96% and a sensitivity of 41% for IPAH vs control. When two markers were above the cut-off, the specificity and sensitivity were 80% and 86% for IPAH vs control. When only one marker was above the cut-off, IPAH was detected with a sensitivity of 100% and a specificity of 60%. For CTEPH, the same marker combination (3 of 3) with adjusted thresholds resulted in a specificity of 100% and a sensitivity of 43%. Two positive criteria were associated with a sensitivity of 78% and a specificity of 88%, and one positive criterion (NT-proBNP >305 pg/ml, or apelin-17 > 1,270 pg/ml, or GDF-15 > 1,220 pg/ml) with a sensitivity of 91% and a specificity of 72% (Table 3).

**TABLE 3:** A combination model for predicting IPAH and CTEPH vs. controls using different cut-offs for IPAH (apelin-17 > 1,480 pg/ml, NT-proBNP >140 pg/ml, GDF-15 > 855 pg/ml) and CTEPH (NT-proBNP >305 pg/ml, apelin-17 > 1,270 pg/ml, GDF-15 > 1,220 pg/ml).

	IPAH		CTEPH	
	Sensitivity	Specificity	Sensitivity	Specificity
**One marker > cut-off**	100%	60%	91%	72%
**Two markers > cut-off**	86%	80%	78%	88%
**All markers > cut-off**	41%	96%	43%	100%

### 3.3 Correlation analysis

In IPAH, apelin-17 was not significantly correlated with demographic data or hemodynamics. In contrast, GDF-15 was positively correlated with PAWP (*ρ* = 0.46), NT-proBNP (*ρ* = 0.56), uric acid (*ρ* = 0.63) and WHO class (*ρ* = 0.57). NT-proBNP was positively correlated with PAWP (*ρ* = 0.48), RAP (*ρ* = 0.64), WHO FC (*ρ* = 0.47), bilirubin (*ρ* = 0.52), uric acid (*ρ* = 0.63) and GDF-15 (*ρ* = 0.56). Moreover, it was negatively correlated with CO (*ρ* = -0.6), CI (*ρ* = -0.65) and 6MWD (*ρ* = -0.42) (Supplementary Table S1 in the Supporting Information).

In CTEPH, apelin-17 was positively correlated (*p* < 0.05) with markers of right heart failure (RAP *ρ* = 0.49, NT-proBNP *ρ* = 0.46), uric acid (*ρ* = 0.46) and bilirubin (*ρ* = 0.44). GDF-15 was negatively correlated with resting arterial pCO_2_ (*ρ* = -0.56) and 6-min walk distance (*ρ* = -0.54). There were also significant correlations with mPAP (*ρ* = 0.44), RAP (*ρ* = 0.44), PVR (*ρ* = 0.4), and NT-proBNP (*ρ* = 0.61) (Supplementary Table S2 in the Supporting Information). NT-proBNP was significantly correlated with mPAP (*ρ* = 0.75), PVR (*ρ* = 0.72), CI (*ρ* = -0.44), 6MWD (*ρ* = - 0.42), pCO2 (*ρ* = -0.59), bilirubin (*ρ* = 0.5), uric acid (*ρ* = 0.44), apelin-17 (*ρ* = 0.46) and GDF-15 (*ρ* = 0.61).

## 4 Discussion

### 4.1 Circulating apelin isoforms

We prospectively investigated the diagnostic value of apelin-17 for the diagnosis of IPAH compared with age-and sex-matched controls and compared this to NT-proBNP and GDF-15, the most established markers of PAH, to better interpret and classify our results. As a main finding, we found that apelin-17 distinguished IPAH from controls with an AUC comparable to NT-proBNP and GDF-15. In addition, we found that our CTEPH cohort, when compared to the controls, was identified by serum apelin-17 with an accuracy comparable to IPAH. This finding suggests that apelin-17 may not only be a marker for IPAH but for pulmonary hypertension, in general. In patients with three positive markers for PH (apelin-17 and GDF-15 and NT-proBNP), the specificity for PH was very high, but at the expense of sensitivity. In patients with at least one positive marker, the sensitivity for PH was 100%, but at the expense of specificity.

Apelin isoforms have been discussed as potential new biomarkers for PH but have not been investigated in a prospective study after a formal power calculation. A previous study by Zhen et al. demonstrated the presence of at least apelin-13, apelin-36 and apelin-17 in the plasma of healthy controls and pyroglutamil apelin-13 was proved to be the major isoform (Zhen et al., 2013). Apelin-12 was rapidly degraded under physiological conditions, consistent with the observation from our pilot study that apelin-12 was detectable in only 2/10 samples.

In our prospective cohort, in contrast to NT-proBNP and GDF-15, apelin-17 was not significantly correlated with any of the pulmonary hemodynamic markers in IPAH. This suggests that it may be independent of the actual cardiac and vascular stress and might rather represent the remodeling activity. However, this is speculative as in CTEPH, apelin-17 was significantly correlated with hemodynamic parameters. These differences in the apelin-17 associations between IPAH and CTEPH could be due to different underlying pathological disease mechanisms or the small sample size.

We did not aim to assess if apelin-17 may be a prognostic marker as well, however it is tempting to speculate that elevated levels may be present in the whole disease course since PH is a progressive vasculopathy. Therefore, future studies assessing apelin-17 as a prognostic biomarker in PH are warranted.

### 4.2 Role of apelin in the circulation

The importance of apelin-17 is supported by *in vitro* evidence that this isoform has the highest affinity for the APJ receptor (Yang et al., 2017b). In addition, there are several studies showing that synthetized apelin-17 analogs have potent effects on the APJ receptor (Iturrioz et al., 2010; Gerbier et al., 2017; McKinnie et al., 2017; Fischer et al., 2020; Flahault et al., 2021). However, only few studies investigating circulating apelin have distinguished between isoforms. Under normoxic conditions, an apelin mixture consisting of many isoforms inhibited the vasoconstriction of pulmonary arteries as shown by wire myography under normoxic but not under hypoxic conditions (Andersen et al., 2009). In another approach, estradiol administration to male SuHx-PH rats decreased RV proapoptotic signaling and RV apelin RNA levels (Frump et al., 2015). Elabela/Toddler is another apelin receptor ligand that showed similar effects to apelin in PH models despite lacking sequence similarity (Yang et al., 2017a). Thus, apelin acts as a vasoactive and likely anti-remodeling mediator in the pulmonary circulation. There are only a few therapeutic approaches, and most of them were made with apelin-13, which improved vascular remodeling in mice exposed to chronic hypoxia (Fan et al., 2020) [pyr^1^]-Apelin-13 infusion decreased PVR and increased cardiac output in an acute study of 19 PAH patients (Brash et al., 2018). This indicates that the upregulation of apelin in pulmonary hypertension represents a negative feedback loop, comparable to natriuretic peptides.

### 4.3 Limitations

As a limiting factor, the number of patients in our study was small, but this was consistent with our power calculation and yielded a highly significant result. At the time of recruitment, PH was defined as mPAP ≥25 mmHg hence we did not include patients who had a mPAP >20 mmHg but less than 25 mmHg. Some of the prospectively enrolled patients had already participated in the pilot study. However, these patients were enrolled in the prospective study on a *de novo* basis and the previous results were blinded to the investigators. Most patients were on targeted PAH therapy and over time, medication changed in several patients. Therefore, it cannot be excluded that medication affected the apelin levels. However, it seems very unlikely that this explains the significant differences between PH patients and controls. We enrolled only patients with severe pulmonary hypertension and healthy controls and found strong contrasts in apelin-17 levels. It is possible that the discriminatory power would be lower in patients with mild PH or other forms of PH. However, the same applies to the established markers NT-proBNP and GDF-15, which did not perform better than apelin-17 as biomarker for PH.

## 5 Conclusion

Based on this prospective study, apelin-17 is a diagnostic marker for IPAH with a diagnostic performance comparable to the established biomarkers NT-proBNP and GDF-15. The study suggests that this is not specific for IPAH but may be also true for CTEPH.

## Data Availability

The original contributions presented in the study are included in the article/Supplementary Material, further inquiries can be directed to the corresponding author.
