# Supplementary material for: Apelin-17 to diagnose idiopathic pulmonary arterial hypertension: A biomarker study
Source: Front Physiol. 2023 Jan 4;13:986295. doi: 10.3389/fphys.2022.986295 (PMC9846527; doi:10.3389/fphys.2022.986295)
Supplement: Supplementary file 1 [file DataSheet1.pdf]

Supplementary Material

Figure S1

Serum levels of apelin-13 (A), apelin-36 (B), Apelin-17 (C) and GDF-15 (D) in n=10 IPAH patients, n=10 age- and sex-matched controls as well as n=10 CTEPH patients represented as Whisker-Blot plots. Points above or below the plots represent outliers. p<0.05 was considered statistically significant

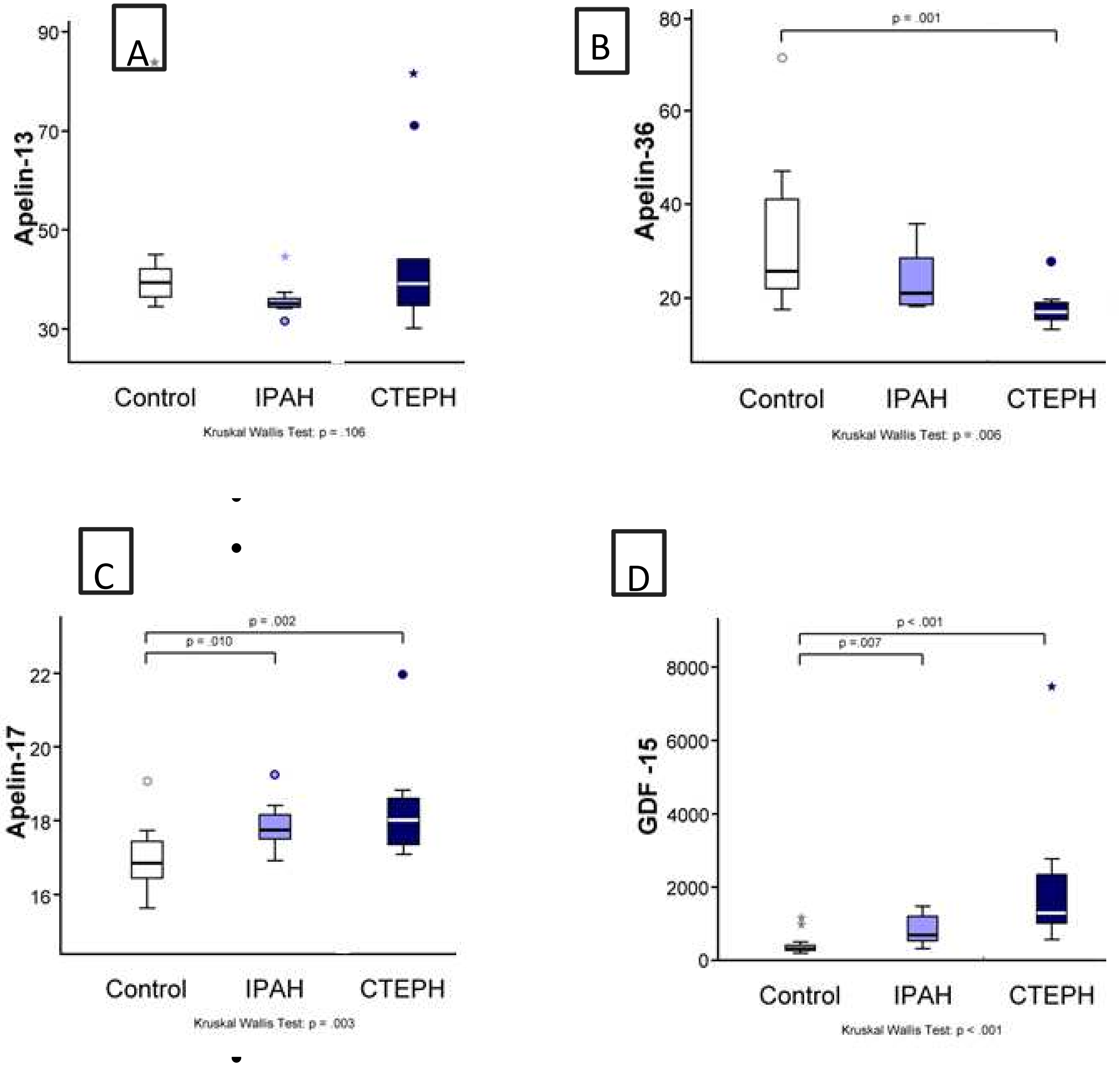

Figure S2

Serum levels of apelin-13 (A), apelin-17 (B), apelin-36 (C) and GDF-15 (D) in n=21 IPAH patients, n=21 age- and sex-matched controls as well as n=21 CTEPH patients represented as Whisker-Blot plots. Points above or below the plots represent outliers. p<0.05 was considered statistically significant

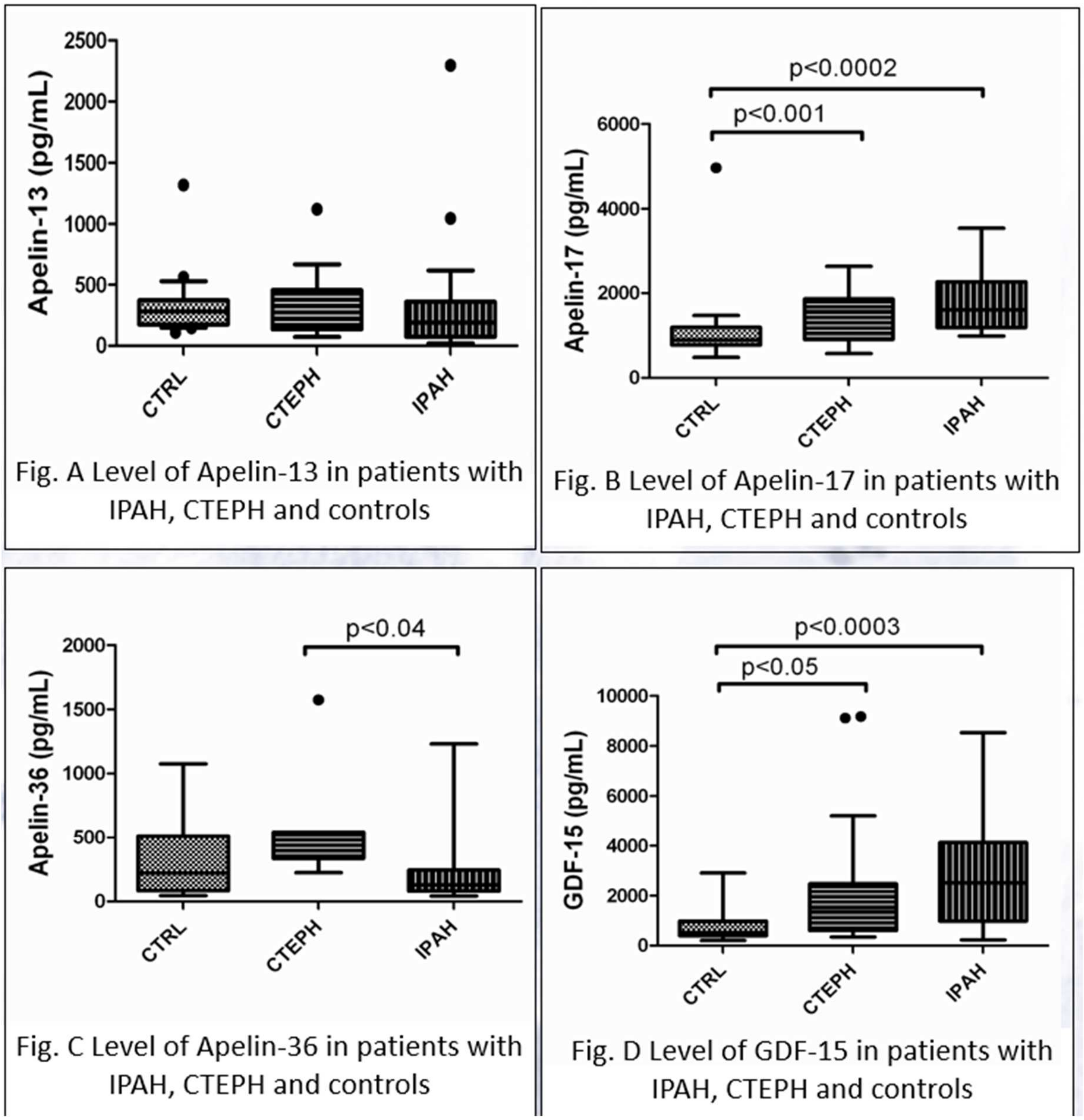

Fig. A Level of Apelin-13 in patients with IPAH, CTEPH and controls

Fig. B Level of Apelin-17 in patients with IPAH, CTEPH and controls

Fig. C Level of Apelin-36 in patients with IPAH, CTEPH and controls

Fig. D Level of GDF-15 in patients with IPAH, CTEPH and controls

Table S1. Spearman correlation analysis results for IPAH patients between the investigated biomarkers and clinical variables. Cells filled with green indicate a significant ($p<0.05$) correlation.

	Apelin-13	Apelin-17	Apelin-36	GDF-15	NT-proBNP
FVC	0,1549	-0,3208	-0,1538	-0,0065	-0,1247
FEV1	0,0773	-0,1898	-0,3494	-0,2965	-0,1949
FEV1/FVC	0,1234	0,2084	-0,4451	-0,7504	-0,4274
HR	0,4638	-0,0203	0,2700	-0,2692	-0,0431
mPAP	0,2721	-0,0547	-0,3778	0,2020	0,3423
PAWP	-0,1887	-0,1530	0,1967	0,4604	0,4754
RAP	-0,2244	-0,2962	-0,0866	0,3470	0,6403
CO	0,2885	-0,1571	0,1813	-0,3854	-0,6008
CI	0,2490	0,0717	0,3242	-0,4383	-0,6464
art. pO2	0,4278	-0,3362	0,7400	0,0514	-0,1627
art. pCO2	0,1790	0,1344	-0,0549	0,2204	-0,0613
PVR	0,1344	0,0159	-0,5000	0,2888	0,3108
6MWD	-0,0974	0,0827	0,0979	-0,3978	-0,4161
WHO FC	0,1727	-0,1028	0,1024	0,5744	0,4685
CRP	0,2610	-0,3020	0,4396	0,3678	0,3228
Bilirubin	-0,0956	-0,0015	0,0524	0,1276	0,5189
Uric acid	-0,0396	-0,0504	-0,0331	0,6325	0,6291
Apelin-13		-0,4839	0,5934	0,0429	0,1401
Apelin-17	-0,4839		-0,5165	-0,2480	-0,2330
Apelin-36	0,5934	-0,5165		0,3077	0,2473
GDF-15	0,0429	-0,2480	0,3077		0,5557
NT-proBNP	0,1401	-0,2330	0,2473	0,5557	

Table S2. Spearman correlation analysis results for CTEPH patients between the investigated biomarkers and clinical variables. Cells marked with green indicate a significant ($p<0.05$) correlation.

	Apelin-13	Apelin-17	Apelin-36	GDF-15	NT-proBNP
FVC	-0,0313	-0,0435	0,0714	0,0400	-0,2126
FEV1	-0,0905	0,0948	0,4643	0,0448	-0,1260
FEV1/FVC	-0,2548	0,1904	0,4286	-0,3635	-0,2095
HR	0,2258	-0,2606	-0,5586	0,0492	0,1360
mPAP	0,2488	0,3900	-0,2342	0,4588	0,7538
PAWP	0,1155	0,1762	-0,5189	0,2500	0,3530
RAP	0,1827	0,4983	-0,1112	0,4786	0,7827
CO	-0,1661	-0,0583	-0,5714	-0,2670	-0,3617
CI	-0,1300	-0,1248	-0,5714	-0,2531	-0,4379
art. pO2	-0,2706	-0,1518	0,6429	-0,3423	-0,2561
art. pCO2	-0,3080	-0,3241	-0,1071	-0,5563	-0,5896
PVR	0,2548	0,2730	0,7500	0,4052	0,7283
6MWD	-0,1720	-0,1621	-0,6000	-0,5377	-0,4236
WHO FC	0,2806	0,1196	0,1443	0,1423	0,0678
CRP	-0,1858	-0,0104	-0,5357	0,2724	0,0064
Bilirubin	0,2614	0,4418	-0,0357	0,3092	0,5056
Uric acid	-0,0309	0,4624	-0,1429	0,3393	0,4390
Apelin-13		-0,2661	-0,4286	0,4209	0,2105
Apelin-17	-0,2661		0,1786	0,3791	0,4634
Apelin-36	-0,4286	0,1786		0,3571	0,3214
GDF-15	0,4209	0,3791	0,3571		0,6166
NT-proBNP	0,2105	0,4634	0,3214	0,6166	

Abbreviations: FVC: forced vital capacity, FEV1: forced expiratory volume in the first second, FEV1/FVC: Tiffeneau index, HR: heart rate, mPAP: mean pulmonary arterial hypertension, PAWP: pulmonary arterial wedge pressure, RAP: right atrial pressure, CO: cardiac output, CI: cardiac index, art. pO2: arterial partial pressure of oxygen, art. pCO2 :arterial partial pressure of carbon dioxide, PVR: pulmonary vascular resistance, 6MWD: six-minute walking distance, WHO-FC: World Health Organization Functional Class, CRP: C-reactive protein.
